# Open Access to the Scientific Journal Literature: Situation 2009

**DOI:** 10.1371/journal.pone.0011273

**Published:** 2010-06-23

**Authors:** Bo-Christer Björk, Patrik Welling, Mikael Laakso, Peter Majlender, Turid Hedlund, Guðni Guðnason

**Affiliations:** 1 HANKEN School of Economics, Helsinki, Finland; 2 Innovation Center Iceland, Reykjavik, Iceland; University of East Piedmont, Italy

## Abstract

**Background:**

The Internet has recently made possible the free global availability of scientific journal articles. Open Access (OA) can occur either via OA scientific journals, or via authors posting manuscripts of articles published in subscription journals in open web repositories. So far there have been few systematic studies showing how big the extent of OA is, in particular studies covering all fields of science.

**Methodology/Principal Findings:**

The proportion of peer reviewed scholarly journal articles, which are available openly in full text on the web, was studied using a random sample of 1837 titles and a web search engine. Of articles published in 2008, 8,5% were freely available at the publishers' sites. For an additional 11,9% free manuscript versions could be found using search engines, making the overall OA percentage 20,4%. Chemistry (13%) had the lowest overall share of OA, Earth Sciences (33%) the highest. In medicine, biochemistry and chemistry publishing in OA journals was more common. In all other fields author-posted manuscript copies dominated the picture.

**Conclusions/Significance:**

The results show that OA already has a significant positive impact on the availability of the scientific journal literature and that there are big differences between scientific disciplines in the uptake. Due to the lack of awareness of OA-publishing among scientists in most fields outside physics, the results should be of general interest to all scholars. The results should also interest academic publishers, who need to take into account OA in their business strategies and copyright policies, as well as research funders, who like the NIH are starting to require OA availability of results from research projects they fund. The method and search tools developed also offer a good basis for more in-depth studies as well as longitudinal studies.

## Introduction

### The emerging phenomenon of Open Access

During the past two decades, scientific journal publishing has undergone a veritable revolution, enabled by the emergence of the World Wide Web. This revolution contains two interconnected phases. The first, and to date most visible, is the rapid shift from print only journals to parallel print and electronic publishing [1]. Ten years ago scholars and scientists did almost all their reading from paper journal issues, obtained as personal copies, circulating inside their organisations, or by retrieving the issues from library archives. Today the predominating mode is to download a digital copy and either read it directly off the screen or as a printout. This has been facilitated by publishers' electronic licensing to bundles of journals (“big deals”) and awareness tools such as emails containing tables-of-content of new issues of favourite journals. Today the average researcher at a university has instant access to a much broader range of journal articles than ever before during the print era.

The second stage in this revolution is access to articles without any restrictions posed by subscriptions, commonly referred to as Open Access [2]. Open Access emerged in the early 1990s, triggered by the possibilities offered by the web, but also partly as a reaction to the so-called “serials crisis” [3] of subscription prices, which seemed to be constantly rising faster than the rate of inflation. In the early days most Open Access journals were small-scale individual operations run by groups or individual scientists, much in the same spirit as Open Source Software projects [4]. After the year 2000 an increasing number of professional Open Access publishers have emerged. (i.e. BioMedCentral, Public Library of Science, Hindawi, Bentham Open). These publishers typically finance their operations by publication charges levied on the authors of the articles, reversing the business model from being content sellers to being dissemination service providers, making the authors their clients rather than the readers. Today the number of OA peer reviewed journals is around 5000 (well documented in the Directory of Open Access journals, DOAJ). In addition to journals which are fully 100% Open Access, there are other journals which operate via subscriptions as mainstream journals do, but which offer open access to the electronic versions of their articles after a delay of usually a year, or selectively for individual articles provided the authors have paid an additional charge to “open up” the articles.

Open Access journals provide one solution to the problem of restricted access to results of publicly funded research. The other is supplementing the dominant subscription-based literature by free copies of the manuscripts, posted by the authors or their institutions on different types of web sites. In the early days the home pages of the authors or their departments was the typical place, and often the only place, to put such copies. Today digital copies are increasingly posted in subject-specific repositories such as the renowned arXiv, which started out focused on physics papers but has since expanded its disciplinary scope, or alternatively in repositories maintained by individual universities for providing archiving and access to the output of their faculty. A majority of international publishers actually allow the posting of some versions of published articles, sometimes after a delay, in such repositories. This latter solution to the access problem is often by OA activists called the “green route” as opposed to the “gold route” of direct OA journal publishing [5]. Green copies come in a number of variations of decreasing value to the readers. The most useful ones are direct digital copies or scanned-in versions of the articles as published [6]. Most publishers prefer to allow posting of the authors manuscripts after acceptance for publication, but before final copy-editing and pagination. The author manuscripts as originally submitted for peer review differ the most from the final published articles. In a few scientific disciplines, such as physics or economics, there are long-standing traditions of circulating such copies widely via preprint servers, or as so-called working papers [7].

Over the past fifteen years there has been a lot of debate about the economics of OA versus subscription based publishing [8], as well as about the advantages and disadvantages of gold OA publishing versus green parallel publishing. Proponents have emphasised the direct cost savings that can be obtained by OA in the publishing system and also the positive indirect effects on R&D thanks to increased access [9]. There have also been several studies showing that openly available articles are cited more by peers [10], [11], [12], which provides a strong incentive for authors to post green copies. Opponents have warned of possible dangers to the peer review process and its level of quality control if publishers are forced to move to OA.

A central question many policymakers ask is consequently how common Open Access is today and how fast the share of OA is increasing? What proportion of journal articles are OA and to what extent do researchers post OA copies in repositories? Accurate answers to such questions would be very valuable for instance for research funders, university administrators and publishers. The purpose of the study reported on in this paper is to provide answers to this type of questions.

### Earlier research

Although some estimates of OA prevalence have been published over the last few years, there is a clear need for rigorously conducted and up-to-date studies. So far the volume of OA has been studied for instance in the following ways.

- For gold OA publishing it has been possible to establish an overall share of OA journals by comparing the number of OA journals listed in the DOAJ index to the total number of active peer reviewed scholarly journals listed in the Ulrich's Periodicals directory.
- For green OA there are directories (DOAR, ROAR) listing repositories and statistics of how many documents these contain.
- For particular limited disciplines it is possible to take the content in a few leading journals and check the availability of OA copies using web search robots and manual checking for full-text copies [13].
- Broader studies can be conducted using discipline-specific or global samples using article titles taken from indexing services (ISI, Scopus or Pubmed) which are then searched for using popular web search engines [14].
- For larger masses of articles the availability of full text versions OA can be checked by web crawling robots [10] that are fed by article titles from indexing services.

All these methods suffer from limitations. On average OA journals publish far fewer articles per annum than subscription based ones [15], and thus the share of OA articles in the total global article volume is much lower than the share of titles. The criteria for inclusion in DOAJ and Ulrich's might also differ, so that the number of journals may not be directly comparable. The share of existing OA journals which have been reported in DOAJ has also changed over time. Counting the number of documents in repositories may tell a lot about the growth of the repositories, but the numbers cannot usually easily distinguish between copies of articles published elsewhere and a wide range of other materials (theses, working papers, research data, teaching material etc). The OA figures obtained for a few select narrow disciplines are interesting but don't give the broad picture. Often many journals even in the said disciplines are not included in the sample. This method also works better for green copies than for gold OA. Web Robots offer a very cost-effective way of identifying copies but are prone to mistakes of many sorts, and it is very difficult to classify the found copies into types. The most precise and comprehensive method is manual checking of titles obtained from general indexing services. The downside of this method is that the amount of work is considerable and increases in direct proportion to the number of articles in the sample.

### Aim of this study

Our objective in this study was to make a rigorous assessment of the overall share of the peer reviewed article literature, which is available as OA, either published directly or made available as copies in different sorts of repositories. Furthermore, the variations in the OA availability based on the scientific discipline was also of interest, as well as the breakdown of the available OA copies into types of gold or green publishing and also based on the quality of the copy for green.

## Materials and Methods

### Research set-up

Our methodology was based on making random samples of articles and then testing for the availability of OA copies using the most widely used web search engine (Google). The research set-up was in line with an earlier study we carried out in the winter of 2008 [15], but was for this study more systematic and more comprehensive.

The central research question was: Given a set of peer reviewed journal articles fulfilling some given criteria, what proportion can be found openly on the web, either published directly in open access journals, in journals practicing delayed or selective OA publishing, or via copies posted on the web.

Central to our research design was measuring OA prevalence on an article basis, not on calculating the share of journals which are OA. In the first instance the set of journal articles comprised all peer reviewed articles published globally in a given recent year. Obviously the definition of a scholarly journal article lacks in clarity, but usually there is an assumption that the article reports on original research, has undergone anonymous peer review of some sort, is shorter in length compared to monographs and is published in an issue of a regularly appearing serial publication. For practical reasons we have to use the lists of journals provided by indexes such as Ulrich's, Web of Science, Scopus and DOAJ as a basis for any comparison. These indexes do not have 100% coverage of all the journals that would fit our definition, especially for journals published in other languages than English, but the task of finding data is simplified enormously.

### Sources of data on journals and articles

A prerequisite for carrying out this type of study is the availability of information on the web, either available freely or via subscriptions. A second important border condition is the availability of information in indexes of different sorts which facilitate and speed up the analysis. Firstly, this concerns in particular basic information about journals, and secondly meta-information about scholarly articles.

Our main data sources have been the following:

- Ulrich's Periodicals Directory is the standard source of information about periodicals of all sorts, containing basic information about more than 200000 journals. Using its search features it is possible to extract data about approximately 25000 journals meeting the requirements of scholarly/academic, active and refereed.
- ISI's Web of Science contains bibliographic information about all the articles published in around 8000 peer reviewed journals. Although only about one third of the journals in Ulrich's are indexed by the ISI, the coverage of the number of articles published is much bigger, since Web of Science includes most high quality and high volume journals [15]. An important add-on to the Web of Science is the Journal Citation Reports database, which calculates so-called impact factors based on the citations in the Web of Science. These are widely used in academia as a proxy for the scientific quality of journals.
- Scopus is a rather new service produced by Elsevier, which offers the same types of features as Web of Science. For our purposes Scopus is very useful since its coverage of journals (around 15000) is more comprehensive than that of the Web of Science. Additionally, SCImago is a free service on the web, which based on data in Scopus calculates citation indexes for journals in the much the same way as JCR (Journal Citation Reports).
- The Directory of Open Access Journals (DOAJ) contains basic information about Open Access scholarly journals. It currently contains information about almost 5000 journals, of which around 2/3 are also included in Ulrich's.

Early on in the project a decision was made to construct a master journals database, using relevant information extracted from all the aforementioned sources. Criterion for inclusion was that the journal was active and peer reviewed, and that it was listed in at least one of the four databases (Ulrich's, JCR, Scopus, DOAJ). Using these principles, a database containing information in excess of 30000 journals was built. For about half the journals, those indexed in Scopus, the database also included information about the number of articles published per year as well as measures of the frequency with which articles in the journal are cited in other journals.

### Creating the samples of articles

The base year, which we studied, was 2008. A delay of slightly over one year after publication was important because many publishers using delayed OA use a 12 month embargo period. Also some publishers allowing green OA posting have a one year delay. We did the majority of the article searching during September–October 2009.

The full body of literature of interest consisted of all the peer reviewed articles in all the different fields of science. In constructing the samples there were two contradictory considerations; getting big enough samples for reasonable statistical significance, and keeping the sample sizes small enough to save time in the time-intensive article searches. An additional constraint was that the indexing services used are set up in such a way that the number of downloadable search result items is severely limited, and no assurance of the randomness of the results which are output can be given.

For the purpose of this study, the problem of obtaining random samples was solved by using the advanced search facility of Scopus, which allows searching for articles based on the first or the last page number of the article. One problem with the samples we obtained in this way, is that journals publishing issues that always start from the number one, rather than having numbering that continues throughout the year, would have a higher probability of being included. In addition some journals may have several volumes in the same year. Also some rare journals might have multiple short articles on the same pages. One way to avoid the first problem was to choose relatively high numbers (over 100) as parameter values. On the other hand the page numbers used cannot be much higher than 100 since this would imply a clear bias towards high volume journals. Due to the fact that some articles might be classified under multiple subject areas (a feature of Scopus), we used different first pages for different subjects, to avoid the same article accidentally being included in several samples. In the end we obtained a few journals with multiple articles in the samples. Duplicate journal entries were deleted so that each journal only was represented once in resulting sample.

Since each journal consequently had an approximately equal chance to get into the sample these do not well represent the population of articles as a whole. To compensate for this we weighted the results with the yearly number of articles published by the journals in question, this data could be found for almost all journals in the SCImago database. Thus the adjusted results are representative of the overall mass of articles. Provided that sufficiently large samples were constructed, this should lead to getting approximately the same results as by having a fully randomised sample of articles from the start. But with relatively small samples the influence of a few sampled articles from big volume journals with over 1000 articles published per year became disproportionate. For this reason, additional articles (10 articles each, given 1/10 weight each) were included for the 26 largest volume journals so that more reliable results, particularly concerning the availability of green copies from the journals in question, could be obtained.

The differences between disciplines were handled by construction of sufficiently large samples of articles for each scientific discipline using the Scopus breakdown. Scopus was first queried for a distribution of the articles published in 2008 according to discipline. Since the database allows the classification of individual articles (or journals) as belonging to several disciplines at the same time the total number of articles obtained via discipline-specific searches (1.97 M) exceeds the number of unique articles (1.27 M) by 54%. Despite this we felt that we could use this split to determine the overall proportions of the global article output by discipline.

Scopus has a breakdown of 28 disciplines, some of which are rather small in their overall article counts. For our purposes we merged some of the categories, for instance several small subdisciplines of medicine. Our aim was to obtain large enough samples for each of our disciplines to make meaningful comparisons across disciplines. In order to obtain samples of approximately equal sizes we varied the number of allowable first pages from discipline to discipline. Our grouping of disciplines and the sample sizes we obtained are shown in table 1.

**Table 1:** Bundles of subject areas and the corresponding sample sizes.

	Scopus Hits	%	% (our bundle)	Sample Size
**Mathematics**	63011	3,2	3,2	194
**Medicine**	366968	18,6	18,7	321
**Areas related to medicine**	198512		10,1	197
* Immunology and microbiology	48062	2,4		
* Pharmacology, Toxicology and Pharmaceutics	46992	2,4		
* Neuroscience	40649	2,1		
* Nursing	19928	1		
* Health professions	19027	1		
* Veterinary	14921	0,8		
* Dentistry	8933	0,5		
**Biochemistry, genetics and molecular biology**	174803	8,9	8,9	207
**Chemistry and chemical engineering**	190077		9,7	169
* Chemistry	129276	6,5		
* Chemical Engineering	60801	3,1		
**Physics and astronomy**	160547	8,1	8,2	182
**Engineering in broad**	377698		19,3	209
* Engineering	170567	8,6		
* Materials science	121671	6,2		
* Computer Science	56687	2,9		
* Energy	28773	1,5		
**Earth and Environmental Sciences**	240309		12,3	206
* Earth and planetary sciences	62886	3,2		
* Environmental Science	62733	3,2		
* Agricultural and biological sciences	114690	5,8		
**Social Sciences, Arts and Humanities**	189194		9,7	152
* Arts and Humanities	22715	1,2		
* Business, management and accounting	28196	1,4		
* Decision Sciences	10363	0,5		
* Economics, Econometrics and Finance	19563	1		
* Psychology	31377	1,6		
* Social sciences	76980	3,9		
			100,0	1837

There are two larger disciplines, Medicine and “Engineering in broad”. Areas related to Medicine could equally well have been merged with Medicine. Mathematics is a rather small area, but difficult to group with others. We kept it separate because of previous knowledge that it is rather interesting from an OA perspective.

Since the searching for OA copies and establishing the type of the found copy is very labour-intensive, we decided to use the results obtained using the discipline-specific breakdown to also construct the global averages. We could not just merge the samples into one, since smaller disciplines would have become overrepresented. Instead, we used the proportions of the disciplines in the overall output of 2008 (see table 1) as weights in calculating the overall averages.

### The search process

The logical way of researching the OA prevalence is to do searches in some web search engine based on the titles of individual published articles. This in fact mimics what most readers would do if they find an interesting citation to an article, but without a direct hyperlink to click on. Other researchers have used a combination of different search engines (Google, Google Scholar, OAIster etc). Norris, Oppenheim and Rowland (2008) [11] tested the coverage of Google, Google Scholar, OAIster and OpenDOAR for finding copies and reported that 86% of the copies could be found using either Google or Google Scholar. For reasons of economy we chose to use only Google. Thus we feel that our method answers the question “what share of OA copies would the average researcher find” rather well, compared to the alternative “what share of OA copies are available somewhere on the web”.

Since several people took part in the searching we were concerned about the integrity and uniformity of the collected data. As a consequence, an easy-to-use data collection tool was developed in the Visual Basic for Applications macro language.

**Figure 1:**
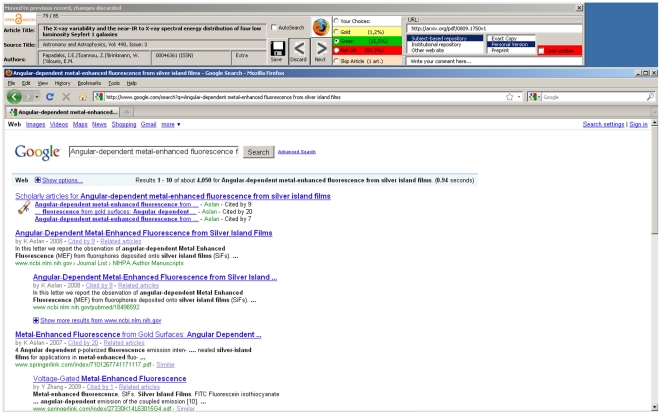
Screenshot of the search tool.

The main functionality consisted of linking the web browser with the bibliographical data so that an automated web search could be triggered by selecting a specific article in the search tool. An important consideration was to have the tool take up as little screen space as possible in order to avoid flipping back and forth between windows, a screenshot of the tool together with an ordinary web browser can be seen in figure 1. Another feature was automated data validation as the classification of articles was done through a graphical user interface with predefined choices and functionality. The usage of such a tool both speeded up the time-consuming task of searching and classifying the results and also improved the consistency of the searching.

The search term was the full title of the article, and clicking through results restricted to the first ten hits on Google. Our experience indicated that if the article was to be found at all, it usually showed up in the 5–6 first hits and the later search results were usually references to the original article included in other articles.

Most of the articles were only accessible via subscription, and in some cases an article was not accessible at all due to the journal having no online content. These articles were classified as not found. Some cases for which open full texts were found they were discarded from the sample altogether, if it was obvious that the item was not a peer reviewed scholarly article or that it was misclassified by the source database and clearly belonged to another discipline.

For original articles found at the publisher's official site or at a site like High-Wire Press, which hosts the electronic versions of journals, we classified the article into direct OA, delayed OA and article-specific OA. In some cases articles were found which were OA on subscription sites, but which were sample free copies or openly accessible only by accident, clearly against the site policy. We classified these as not found, since we were only looking for individual articles which if OA were likely to stay so for the foreseeable future. This is the case for paid OA in schemes such as Springer's Open Choice and Oxford Open. In some cases several hits to OA full text versions were found. We instructed the searchers to include information about only one copy, in the following order of preference if several were found: OA journal site, Subject-based repository, Institutional repository and Other web site.

We also studied the type of copy found in the repositories and on other web sites. In addition to direct copies of the article as published, a separate category was identified as so-called authors manuscripts which are accepted for publishing, but still need to have the final copyediting and page layout completed (“personal versions”). “Preprint” versions which are earlier versions of a submitted manuscript, were also accepted since they are considered very important in some fields of science (for instance working papers in economics). A preprint version was accepted only if it had exactly the same title as the published article. Often conference articles and working papers are earlier versions of later peer reviewed and published articles, but use slightly different titles.

The articles in each sample file were searched for and the results were classified by one researcher. After that the findings were cross-checked by another researcher (often the research leader), with focus in particular on the classifications of found OA copies. This led to only minor changes in the observations.

### Statistical reliability of the results

As reported above our method was not a fully random sampling method with the disciplines. Due to the complexity of the method it is not possible to calculate exact confidence intervals for the OA-shares. What we can do is to calculate confidence intervals for a few of our results, under the assumption that we had been able to use fully random samples, thus ignoring the complexities of the first page search and normalising by journal size. In the following table we have calculated confidence intervals for 95% confidence level for a few cases.

We note that the *margin of error* is defined as the radius (half of the width) of the confidence interval. Denoting the margin of error by *c*, we can compute an estimate for the *minimum* margin of error that can be attained under a fixed sample size *n*, estimated value *p*, and level of confidence 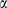 by the following formula
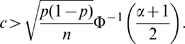
where 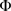 denotes the standard normal (cumulative) distribution function, i.e.
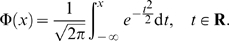
The calculations in table 2 show that we can talk with reasonable confidence about the global OA share. The results concerning differences between disciplines as well as the results concerning gold and green shares or split downs in green types have to be interpreted with much caution.

**Table 2:** Statistical reliability.

Parameter	Sample size (*n*)	Estimated value (as %) (*p*)	Minimum margin of error (as %) (*c*)(*a* = 95%)	Confidence interval (in %)(*a* = 95%)
**Overall OA**	1837	20.4	1.84	(18.56, 22.24)
**Overall gold**	1837	8.5	1.28	(7.22, 9.78)
**Overall green**	1837	11.9	1.48	(10.42, 13.38)
**Overall OA, medicine**	321	21.7	4.51	(17.19, 26.21)
**Green OA, Mathematics**	194	17.5	5.35	(12.15, 22.85)
**Institutional repository copies**	1837	2.9	0.77	(2.13, 3.67)

## Results

### Overall OA-shares

The weighted average OA availability over all disciplines was 20,4%. This further splits up into 8,5% in OA journals and 11,9% copies in repositories and web sites.

Since many previous and parallel studies have been based on samples from ISI indexed journals, we tested our results by dividing our material into two groups: Articles indexed in ISI journals, and articles not indexed in ISI (but found in Scopus) ‘

For practical reasons we used the subject classifications derived from Scopus to weight the results. The results are shown in table 3.

**Table 3:** Overall gold and green prevalence for ISI and non ISI articles.

	No of articles	Gold OA	Green OA	Total OA
**ISI journal articles**	1282	6,6	14,0	20,6
**Non-ISI articles**	555	14,2	5,5	19,7
**All articles**	1837	8,5	11,9	20,4

The overall OA-results are relatively similar for the ISI and non-ISI subsets. The results indicate also that the proportion of gold OA is clearly lower in the ISI subset. This could be explained by the fact that it has been more difficult for relatively new journals (which is the case for most journals born OA) to get accepted into ISI, than into Scopus. On the other hand, the proportion of green copies is much higher in the ISI subset. A plausible explanation could be that authors are more likely to put copies of their higher quality articles in repositories. [16] term this the “selfselection bias”.

**Figure 2:**
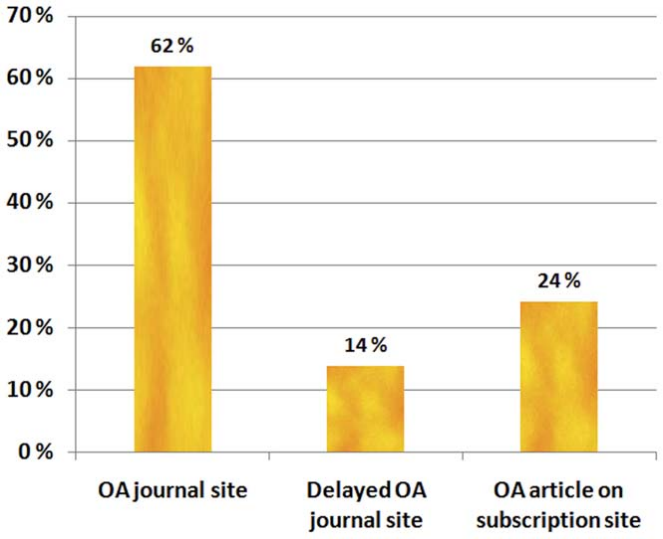
Split of found OA journal articles into types.

The split of the OA journal articles into categories is shown in the figure 2 below.

**Figure 3:**
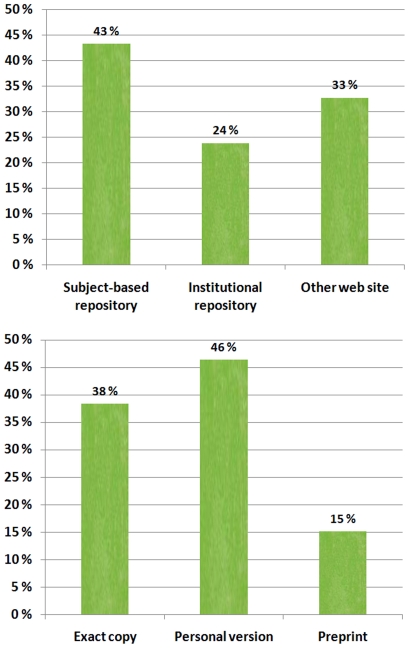
Breakdowns of green OA copies.

For green copies there are two breakdowns of interest, the type of repository and the type of copy. These are shown in figure 3.

In the subject-based repository category the most frequently encountered repositories were arXiv and PubMedCentral. Institutional repositories were of an archival calibre, often implemented by using either the D-Space or the E-prints software. Copies of papers uploaded by the authors to web pages at their own departments, often using non systematic addressing, were classified as other web site. Typically, this kind of web pages were authors' homepages consisting of for example CVs and links or samples of published research papers.

### OA availability by scientific discipline

**Figure 4:**
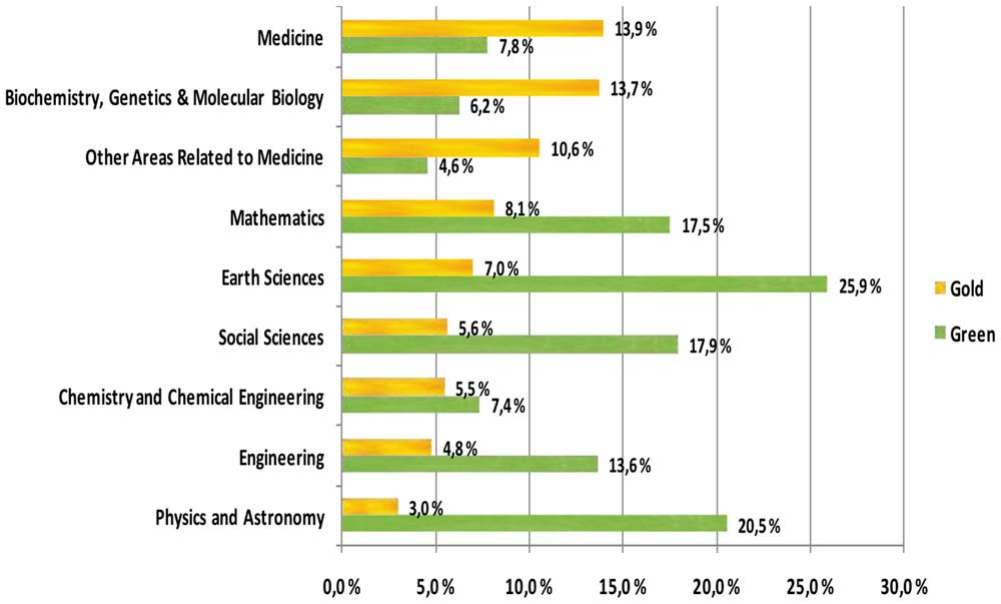
OA availability by discipline.

The availability of gold and green OA copies by scientific discipline are shown in figure 4. The disciplines are shown by the gold ratio in descending order, rather than in alphabetical order.

There is a clear pattern to the internal distribution between green and gold in the different disciplines studied. In all the life sciences, gold is the dominating OA access channel. The picture is reversed in the other disciplines where green dominated. The lowest overall OA share is in chemistry with 13% and the highest in earth sciences with 33%.

## Discussion

Our results concerning the overall OA share are well in line with the results of the study we did of 2006 articles [15]. In that study we concluded, using different methods and a much smaller sample, that there were 8,1% gold OA articles and 11,3% green copies. The overall share of OA was 19,4% compared to the 20,4% in this study. The difference can be explained by a number of factors.

- The difference is within the confidence interval
- The two studies used partly different methods
- The share of OA is changing with time and two years had lapsed between the studies.

The most thorough study we have found to compare our results with is Matsubayashi et al (2009) [14]. Using methods similar to ours they studied the OA availability of articles in biomedicine from 2005. Their source of article metadata was the Pubmed bibliographic database.

Their material included both peer reviewed articles and news items etc. They reported an OA percentage of 26,3 for peer reviewed articles (70% of their sample), and if the overall share of OA articles requiring registration (0,4%) is subtracted the number comparable to our study would be 25,9%.

Due to the high number of articles analyzed, their confidence interval should be rather narrow and their results rather reliable from a statistical viewpoint. Currently Pubmed offers a search facilty in which one search term is “link to free full text”. We did a search for all “journal articles” published in 2005 with no restrictions (636162 hits) and then repeated this with the further restrictions of “link to free full text”. The OA percentages obtained this way were 23,1 for 2005 and 23,3 for 2008. The figure should include all OA journals (full and delayed) as well as full text deposited in Pubmed Central (either as exact replicas of author manuscripts).

It should be noted that Matsubayashi et al (2009) [14] found 72% of their OA copies at journal websites, 26% in PubmedCentral and 17,4% in journal platforms or portal sites (like Scielo). The numbers add up to more than 100% due to possible duplication. Thus their figures are well in line with the above rather exact figures from Pubmed. More so since they estimated that of all the OA copies they found only 5,9% were in Institutional repositories and 4,8% on author's personal websites (which sums up to 2,8% of all analyzed articles).

The difference between their results and our results (In particular our discipline-specific results for medicine, areas related to medicine and biochemistry) could be caused by a number of factors:

- Use of Pubmed vs. Scopus as a source for article data
- Different base year and time delay from publishing to searching for copies
- Different search strategy. We only used Google. Matsubayashi et al used four different databases and search engines to identify full text copies. They also checked the 20 first results in Google and Google Scholar whereas we only checked the first page.
- The method of obtaining the sample (a search based on the pagination of articles) was the same but we compensated for the possible bias towards small journals by multiplying by the number of articles published per year.

In a study concerning the journal output from 2003, Mc Veigh (2004) [17] found that out of 747060 citable articles indexed in the ISI Web of Science 2,9% were in open access journals. This can be compared to our 6,6% of gold OA in ISI journals. It should be noted that our figures also include delayed OA and article specific OA.

Bhat (2009) [18] studied the OA availability of the research articles from 2003–2007 indexed by Scopus by five leading Indian research institutes. Of the 17516 articles studied 7,8% were published in Open Access journals (either full or delayed). About two thirds of these were in Indian Open Access journals. The study did not include green copies.

In a study of the citation advantage of OA Norris, Oppenheim and Rowland (2008) [11] also calculated the OA availability of a 4633 articles from 65 high-impact factor journals (included in Web of Science) in four subjects, Applied Mathematics, Ecology, Economics and Sociology. They specifically recorded only green copies, which had the same title and authors as the published article and discarded any hits to the publisher's web site. The availability of OA copies was very high in Economics (65%), Applied Mathematics (59%) and Ecology (53%) but considerably lower in Sociology (21%). Since the purpose of their study was specifically to study the citation advantage it appears that they have on purpose included subjects which a-priori were known to have a tradition of posting green copies.

Way (2010) [13] studied the OA availability of articles published in 2007 in 20 top journals (using ISI's journal impact factors) in Library and Information Science. The overall OA share was 27% over a sample of 922 articles. Way also classified the green copies and found that subject-based repositories (38%) and personal web sites (29%) were the two most common locations for the copies.

The study with the biggest sample of articles was Hajjem et al (2005) [10] who used web robot techniques to study the citation advantage of OA. They also calculated the OA availability of 1,3 million articles from 1992–2003 in 10 disciplines and found that the overall OA share was between 5% and 16%. These figures are difficult to compare with.

The clear majority gold articles that we found in our study were in pure gold journals (62%). Articles in delayed OA journals only summed up to 14%. Studying the prevalence of delayed OA articles is much more difficult than pure OA ones, since the journals containing the latter tend to be listed in DOAJ, whereas just about the only site where more aggregate information about delayed OA journals can be found is the Highwire Press website, listing around 200 of the journals they host as offering delayed OA.

We found that 24% of gold articles were individually paid OA articles on subscription sites. This seems to be in line with the few reports available on the actual uptake of this option by authors. For instance [19] reported an average uptake in 2007 of 7% for the 65 journals offering Oxford Open. It is also important to note that only a minority of journals currently offer paid article level access. Of the 9500 journals of 22 major publishers 22% offered this option in September 2009 (informal communication, Max Planck digital library). Theoretically, if 22% of the whole volume of articles from 2008 had this option and the average uptake was 10%, this would lead to a figure of 2,2% of the articles. In principle it would be possible to calculate relatively exact numbers by analyzing the tables of contents for the full 2008 volumes of all the journals of the major publishers offering this option, including over a thousand Springer journals. This task would be very tedious and would probably require using a sampling method.

The overall breakdowns of green copies according to type of repository and type of copy should also be of interest. Since the overall “hits” in each category are rather small we decided not to publish the figures per discipline since they would be very unreliable from a statistical point of view. We can just note that in a few disciplines subject-based repositories dominated, in medicine PubMedCentral and in physics arXiv.

It may come as a surprise that only one out of four green copies was found in institutional repositories. A lot of effort has recently been put into starting such repositories and issuing university guidelines encouraging and requiring academics to post copies there. But compared to the leading subject-based repositories these have had a shorter lifespan so far. Other web sites, in particular the authors' home pages were still the most popular places for placing copies (40%).

Morris and Thorn (2009) [20] surveyed the OA-attitudes and behaviour of members of learned and profession societies in the UK in the winter of 2008. Of particular interest are their figures of where those respondents who practiced self-archiving placed the copies. The figures sum up to over 100% but if they are normalized to 100% the answers are 30,2 for institutional repositories, 11,8 for subject-based repositories and 58,0 for author, departmental and other websites. These figures thus differ quite a lot from our findings, but one has to bear in mind that the questions were differently phrased. Also the spread of the respondents over research fields might differ quite a lot compared to our study.

Fry et al (2010) [21] surveyed author attitudes and behaviour of European researchers. Although they received 3136 responses, a high proportion came from the physical sciences and mathematics (56%). They report on the characteristics of the green copies that the respondents had deposited (p. 33). By normalising their figures to 100% we get the following distribution: preprint version (34%), author final manuscript (38%) and publishers' version (28%). The relative popularity of the different types of repositories was; subject-based repositories (46%), Institutional repositories (45%) and other web sites (9%).

The high share of exact copies we found was slightly surprising, considering the types of copyright restrictions the major publishers pose. In fact, a number of clearly illegal copies were found, where the publishers' files had been copied, usually without proper attribution. Usually these were on the authors' or their departments' home pages. It was also very noticeable that preprints were mainly posted in a few disciplines; mathematics, economics and physics in particular. These areas are known to have traditions of making manuscripts available in the form of preprints or working papers [7].

We will not attempt a more detailed discussion about the possible reasons for the differences between disciplines (for good discussions see [7], [22]). Factors which we believe are particularly important include:

- Uneven spread of available OA journals across disciplines
- Unequal possibilities for financing author charges
- Availability of well established subject based repositories in some disciplines
- Traditions of making preprints available in some subjects

All in all we believe our results should be of interest to science policy makers and scientists alike, providing one of the most comprehensive cross-disciplinary OA studies to date. There are numerous ways to extend the method we have used, for instance comparing more in detail the quality of OA articles compared to non-OA articles. A comparison of the OA availability of articles originating from different countries would be of great interest, since OA has been seen as a great way for authors of developing countries to get their research results better known.
